# Species-Specific Traits plus Stabilizing Processes Best Explain Coexistence in Biodiverse Fire-Prone Plant Communities

**DOI:** 10.1371/journal.pone.0065084

**Published:** 2013-05-29

**Authors:** Jürgen Groeneveld, Neal J. Enright, Byron B. Lamont, Björn Reineking, Karin Frank, George L. W. Perry

**Affiliations:** 1 Department of Ecological Modelling, UFZ - Helmholtz Centre for Environmental Research, Leipzig, Germany; 2 School of Environment, University of Auckland, Auckland, New Zealand; 3 School of Environmental Science, Murdoch University, Murdoch, Australia; 4 Department of Environment and Agriculture, Curtin University, Perth, Australia; 5 Biogeographical Modelling, University of Bayreuth, Bayreuth, Germany; 6 Unité de recherche écosystèmes montagnards, Irstea, Grenoble, France; 7 School of Biological Sciences, University of Auckland, Auckland, New Zealand; Cirad, France

## Abstract

Coexistence in fire-prone Mediterranean-type shrublands has been explored in the past using both neutral and niche-based models. However, distinct differences between plant functional types (PFTs), such as fire-killed *vs* resprouting responses to fire, and the relative similarity of species within a PFT, suggest that coexistence models might benefit from combining both neutral and niche-based (stabilizing) approaches. We developed a multispecies metacommunity model where species are grouped into two PFTs (fire-killed *vs* resprouting) to investigate the roles of neutral and stabilizing processes on species richness and rank-abundance distributions. Our results show that species richness can be maintained in two ways: i) strictly neutral species within each PFT, or ii) species within PFTs differing in key demographic properties, provided that additional stabilizing processes, such as negative density regulation, also operate. However, only simulations including stabilizing processes resulted in structurally realistic rank-abundance distributions over plausible time scales. This result underscores the importance of including both key species traits and stabilizing (niche) processes in explaining species coexistence and community structure.

## Introduction

Neutral models have been used to describe coexistence and some aspects of community structure in species-rich plant communities, such as tropical rainforests and fire-prone Mediterranean-type shrublands [1]–[3]. However, it seems obvious that the basic assumption of neutral theories, that all species within a trophic level are ecologically equivalent, is violated in most systems (but see [4]). For example, in Mediterranean-type ecosystems two distinct adaptations to recurrent disturbance by fire can be identified [5]: some species are killed by fire (non-sprouters) and rely on post-fire recruitment from seeds, while others recover vegetatively (resprouters). These two Plant Functional Types (PFTs) defined by their response to disturbance differ in their longevities by at least an order of magnitude [5], [6]; and the outcomes of neutral theory are sensitive to such differences [7]. In contrast to neutral models, niche-based models seek to explain species coexistence on the basis of stabilizing processes [8]. Intraspecific density regulation is a frequently discussed stabilizing process [9], and intense intraspecific competition due to spatial aggregation at the microsite scale [10] and negative density dependence at the seedling stage [11] both highlight the importance of density regulation for coexistence. Trade-offs between different life-history attributes (e.g. competition *vs* colonization) are also often suggested as mediating coexistence in niche-based models [12]. Trade-offs can be equalizing, i.e. resulting in identical establishment rates for all species, or stabilizing, i.e. under particular conditions (that vary through space and time) different species, especially at low densities, will be favoured. Fundamentally, niche-based processes are stabilizing, whereas under equalizing processes species abundances essentially perform a random walk in the state space [8], [9]. The assumptions of perfect equalizing processes are, however, rarely met [8], [13].

Although neutral and stabilizing models appear to contradict each other, marrying them might help to explain the maintenance of species richness at the community scale [14]. While individual species *do* differ in their functional responses to environmental conditions and in their ecological attributes, groups of species within communities may share traits and so be ecologically similar at this level. Thus, species are often grouped into PFTs [15], [16]. It has been shown that both neutral and niche models produce indistinguishable species abundance distributions so long as there is a sufficient number of species in each PFT [17], [18].

At our study site in fire-prone Mediterranean-type shrublands of southwestern Australia, large shrub species are restricted to dunes where deep, unconsolidated sands provide access to soil water through the summer, while the shallow sands of interdunal areas (separating these dune-top communities) support only low shrubs and graminoids [19]. Local communities of large shrubs on the sand dunes are well connected through long-distance dispersal [20] and therefore provide an ideal system to study the maintenance of diversity in a plant metacommunity comprising contrasting PFTs with many species in each category [21]. Here, we implement a simulation model for two PFTs that differ fundamentally in their response to fire: non-sprouters (fire-killed) and resprouters. We compare scenarios in which species are equivalent within a PFT (neutral) and also where species within the same PFT differ in their key demographic properties, reproductive rate and fire survival probability. Reproductive rate can vary markedly between, and more subtly within, these two PFTs, while survival probability also varies within the resprouter PFT [5], [19], [22], [23]. We address the following questions:

1. Can current metacommunity models explain the long-term coexistence of two PFTs (10 000 and 100 000 years)?
2. Do species have to be strictly ecologically equivalent within a PFT to coexist?
3. How important is intraspecific density regulation as a stabilizing process in accounting for species coexistence?

## Materials and Methods

Our metacommunity simulation model was developed for shrub communities growing on the upper slopes and crests of deep sand dunes (sand to 10 m depth) in the Eneabba Plain, 250–330 km north of Perth [24]. We consider two PFTs: fire-killed species (for scenarios with 8 and 132 non-sprouting species) and vegetatively recovering species (for 8 and 132 resprouting species). Parameterisation of the model and the two PFTs is based on the detailed data set available for the population dynamics of large non-sprouting and resprouting, serotinous (i.e. with on-plant seed storage) shrubs in the genus *Banksia* [25]–[27].

**Figure 1:**
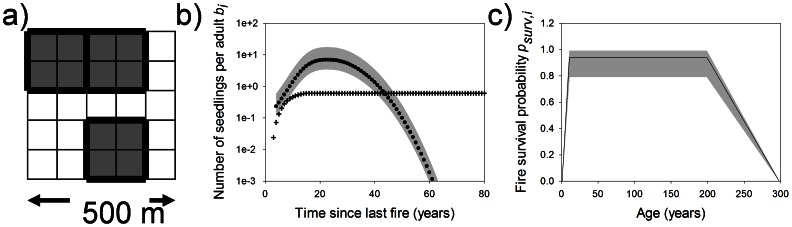
Basic model assumptions. a) The model works at two spatial scales: fire spread is modelled at a 1-ha resolution, whereas neighbourhoods of four contiguous grid cells all containing suitable habitat comprise a patch containing one local community. b) Number of seedlings per adult *b_i_*, vary between mature resprouters (crosses) and non-sprouters (circles). We varied non-sprouter reproductive rates across a wide range of plausible values (shaded grey area in Fig. 1b). c) Fire survival probabilities for resprouters change with plant age; variation in the fire survival probabilities are indicated by the grey shaded area in Fig. 1c.

Our model description follows the ODD (Overview, Design concepts and Details) protocol [28]. Detailed descriptions of the submodels are provided in the electronic supplementary material (ESM, Methods S1). The landscape is represented as a grid of square cells (Fig. 1a). Each grid cell represents 1 ha and we simulate a landscape of 120×120 grid cells, representing an area of 144 square kilometres. Each grid cell contains 65,813 sites (based on density estimates in [3]) that can be either empty or occupied by one individual per site. The metacommunity simulation arena is a square of 40×40 grid cells (16 square kilometres) in the centre of the simulation area. The buffer area minimises edge effects arising in the fire-spread component of the model. Grid cells are classified as being either potential habitat (dunes) or uninhabitable matrix (swales). Habitat islands for large shrubs (patches) comprise four contiguous habitat cells grouped as squares that are distributed randomly so as to occupy half of the metacommunity simulation arena (i.e. 200 patches covering 800 grid cells). To represent spatial heterogeneity in the fire regime, fire spread acts at the grid cell level, so that a patch can either burn entirely or partially (Fig. 1a). At the grid cell level the species identity of a cohort, number of individuals in each cohort, age of cohorts, and the time since last fire are stored.

All species belong to one of two PFTs: non-sprouters or resprouters. Within a given PFT, species may be identical (neutral model), or may vary in their reproductive rate described by number of seedlings per adult *b_i_* (for any species, *i*) (Fig. 1b) and, in the case of resprouters, their fire survival probability, *p_surv,i_* (Fig. 1c). For non-sprouters the reproductive rate *b_i_* is multiplied by a seedling modification factor β_i_ that allows variation in this parameter. β_i_ represents the number of seedlings a non-sprouting shrub has accumulated 9 years after fire. Depending on the scenario intraspecific seedling density regulation is modelled following a logistic model. The grid is updated synchronously at each annual time step. Simulations run for 10 000–100 000 years, corresponding to 200–20 000 generations for non-sprouters, depending on the mean fire interval.

### Process overview and scheduling

The metacommunity model simulates the fundamental demographic processes of birth, death and migration. We explain the fire spread, seed dispersal, seed production, fire survival, and establishment sub-models in detail in the ESM (Methods S1). Depending on the scenario intraspecific negative density regulation of seedlings is considered in the seed dispersal and seed production sub-model. Fire occurs every year (one ignition per year) somewhere on the 144-km^2^ grid, the ignition location (cell) is chosen at random and the fire's spatial structure is determined by a percolation model [29], [30]. Not all fires spread across the metacommunity simulation arena containing the habitat patches. Only burnt grid cells are updated: all plants in burnt habitat patch cells release their (on-plant-stored) seeds, non-sprouters die and resprouters survive with probability *p_surv,i_*. Seedlings can establish in sites that become empty as a result of the fire (depending on inter- and intraspecific seedling competition), but competition from established vegetation prevents them from establishing in unburnt cells. Although we term a site ‘empty’ if none of our study species are in it, we assume that these cells are occupied by species from other PFTs (e.g. sub-shrubs, perennial sedges/rushes). We do not consider seed release in unburnt grid cells, assuming strong serotiny for the modelled species (and no recruitment even if there is any seed release; [31]). Seeds arrive at a site by either local dispersal from a parent plant within the same patch, or via long-distance dispersal from the metacommunity [20]. This distinction between local patches and the metacommunity is similar to the hierarchical design embodied in many neutral models (e.g. [1]), and thus allows comparisons between our approach and neutral models.

### Design concepts

#### Emergence

Community structure is the result of stochastic local events such as establishment and death, and interactions between local communities via long-distance seed dispersal.

#### Interaction

Neighbouring and non-contiguous grid cells are linked by fire, since only burnt grid cells disperse seeds that can potentially establish in other burnt grid cells.

#### Stochasticity

Fire spread, resprouter fire survival, and establishment by lottery competition are represented as random processes.

#### Collectives

Individuals of the same species and same age are modelled as cohorts.

#### Observation

Fire-related characteristics such as mean fire size and local time since last fire are recorded, as are abundances at the metacommunity level. If species differ, their characteristics are also stored. At the end of each simulation, species abundances are stored for each patch.

#### Initialization

All grid cells are initialized with the same number of individuals of each species. The initial local time since last fire is drawn from a uniform distribution in the range 1 to 25 years. The initial age of all non-sprouting cohorts is the same as the local time since last fire. The age of the initial resprouting cohort is drawn from a uniform distribution bounded by the local time since last fire and the maximum longevity of resprouters (c. 300 years; [19]).

### Simulation experiments

We conducted a sensitivity analysis for eight model parameters (Table 1) using a Latin Hypercube sampling design [32]. Parameter ranges were split into 10 intervals. Overall, 201 Latin Hyper Cube samples were taken, resulting in 201×10 = 2 010 parameter combinations. For each combination, we measured the number of non-sprouting species *S_NS_*, resprouting species *S_RE_* and total number of species *S* that survived for 10 000 years. The metacommunity was initialized with 16 (8 non-sprouters and 8 resprouters) species. We explored the effect of each parameter and the average time since last fire in habitat grid cells on the dependent variables (*S_NS_*, *S_RE_*, *S*) using boosted regression trees [33], [34].

**Table 1:** Overview of the model parameters.

Description	Parameter	Reference value	*min*	*max*	Relative nfluence *R_NS_* (%)	Relative influence *R_RE_* (%)
Time since last fire, a function of basic fire-spread probability (*p_c_*) and the mode of fire spread (*f_mode_*)	*t_slf_*				30	42.1
Number of non-sprouter seedlings at age 9	β_i_	1.8	0.5	3.5	32.7	16.4
Maximum fire survival probability	*p_surv,max_*	0.94	0.74	0.99	11.4	21
Range of species-specific variation in *p_surv,max_*	*d_p_*	0	0	0.25	1.7	13
Range of species-specific variation in number of seedlings per adult *b_i_*	*d_R_*	0	0	0.25	13.3	0.1
Strength of density regulation	1/*f_K_*	0	0.1	1	5.2	1.3
Metacommunity dispersal rate	*m*	0.1	0	0.25	4	1.9
Basic fire-spread probability	*p_c_*	0.48	0.47	0.5	1.1	4.2
Mode of fire spread (0: simple percolation; 1: fuel age dependent)	*f_mode_*	0	0	1	0.5	0
Age of maturity for resprouters	*a_2_*	30	-	-	-	
Run time (years)	*t_max_*	10 000	-	-	-	
Demographic threshold	*np*	5.5	-	-	-	
Grid cell size (ha)	*g_size_*	1				
Grid size (grid cells; ha)(total; simulation arena; buffer)	*n_grid_*	14 400; 1 600; 12 800	-	-	-	
Total number of patches	*n_patch_*	200	-	-	-	
Patch area (grid cells; ha)	*A_patch_*	4	-	-	-	
Initial species number for main simulation experiments (total; non-sprouters; resprouters)	*S*, *S_NS_*, *S_RE_*	16; 8; 8	-	-	-	
Maximum number of individuals per grid cell or ha	*K_gridcell_*	65 813	-	-	-	
Parameters of the sigmoidal relation between local time since last fire and fire-spread probability (years^−1^, years);	*d,b*	1; 5				

The sensitivity of the model results for 8 parameters and the average time since last fire was explored using Latin Hypercube sampling, where *min* refers to the smallest possible parameter value and *max* to the largest parameter value. The results of a boosted regression tree analysis are given to indicate the parameters' relative influence on species richness of non-sprouters *R_NS_*, and resprouters *R_RE_*. See text for further details. Parameterisation is mainly based on [25]–[27].

**Figure 2:**
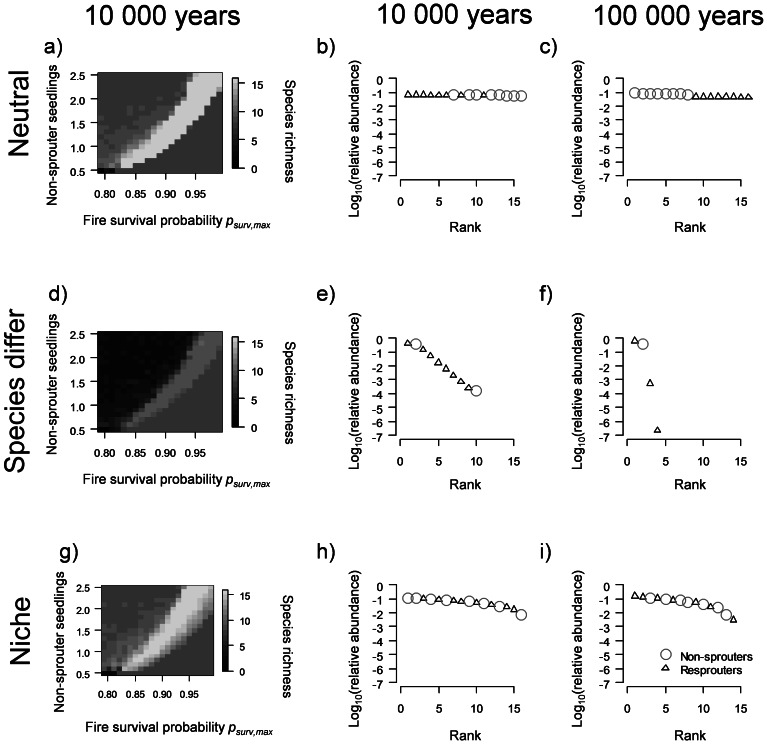
Species richness and RAD. Impact of non-sprouter number of seedlings per adult (the number of non-sprouter seedlings at age 9 β_i_), and resprouter maximum fire survival probability *p_surv,max_* on final species richness (after 10 000 time steps a,d,g) and RAD after 10 000 (b,e,h) and 100 000 years (c,f,i, circles show relative abundance of non-sprouters and triangles relative abundance of resprouters) for three selected scenarios: a,b,c) all demographic parameters are identical for species within a PFT and there is no density regulation (neutral), d,e,f) demographic parameters vary between species within a PFT and there is no density regulation(species differ), and g,h,i) demographic parameters vary between species and density regulation is operating (niche).

We explored three scenarios to investigate how similar species with a given PFT have to be to coexist and to explore the importance of intraspecific density regulation: neutral scenario (no density regulation is applied and all species within one PFT have exactly the same parameterisation – see Table S1 for details), non-neutral scenario (no density regulation is applied and species differ in their species-specific number of seedlings per adult *b_i_* – see Table S1), and niche scenario (density regulation is applied and species differ in their species-specific number of seedlings per adult *b_i_* – see Table S1). To better understand the impact of individual processes and their interactions on species and PFT coexistence, we systematically varied the two key demographic parameters, maximum fire survival probability (*p_surv,max_*) and the number of seedlings for non-sprouters, by varying the number of non-sprouter seedlings at age 9 years β_i_ - for the three scenarios (EMS, Table S1). We used 21 possible parameter values for each of the two parameters (*p_surv,max_* varied over 0.79–0.99 in steps of 0.01 and β_i_ over 0.5–2.5 in steps of 0.125) and simulated all possible (441) pairs. We summed the species richness in the metacommunity, *S_sum_*, for each of the 441 parameter combinations for each scenario (Fig. 2 a,d,g). The fire regime was parameterized as a simple percolation model (*f_mode_* = 0) with a basic fire-spread probability *p_c_* = 0.4825. Finally, we investigated the effect of species richness and time scale on the rank-abundance distributions for the three scenarios with 16 and 264 species (the latter approaches the expected number of species at our study site, after [3]) over 10 000 and 100 000 time steps. For all rank abundance distribution simulations we have parameterized the fire regime as a simple percolation model (*f_mode_* = 0) with a basic fire-spread probability *p_c_* = 0.48.

## Results

### Maintenance of species diversity

A trade-off between fire survival probability and the reproductive rate of non-sprouters has been suggested to be a potential explanation for coexistence between non-sprouters and resprouters [35]. Therefore we systematically varied two key demographic parameters, maximum fire survival probability *p_surv,max_* and the number of seedlings produced by non-sprouters - by varying the number of non-sprouter seedlings at age 9 β_i_ (Fig. 2a,d,g) for the three specific scenarios: 1. If all species within one PFT have the same demographic parameters and there is no density regulation (neutral scenario, EMS Table S1) then species can coexist across a wide range of parameter combinations given a positive correlation between β_i_ and *p_surv,max_* (Fig. 2a, for 21% of all 441-parameter combinations, all 16 species persist). 2. If species differ in their reproductive rates (*d_R_* = 0.1, i.e. species-specific reproductive rates vary ±5% around the mean), but density regulation does not occur (non-neutral scenario, see EMS Table S1), species richness decreases substantially, and for no parameter combinations do all 16 species persist (Fig. 2d). 3. If intraspecific density regulation is in place (*f_K_* = 1) and species differ within each PFT (*d_R_* = 0.1, niche scenario) species coexist across a wide range of parameter combinations (for 13% of all parameter combinations, all 16 species persist) (Fig. 2g).

**Figure 3:**
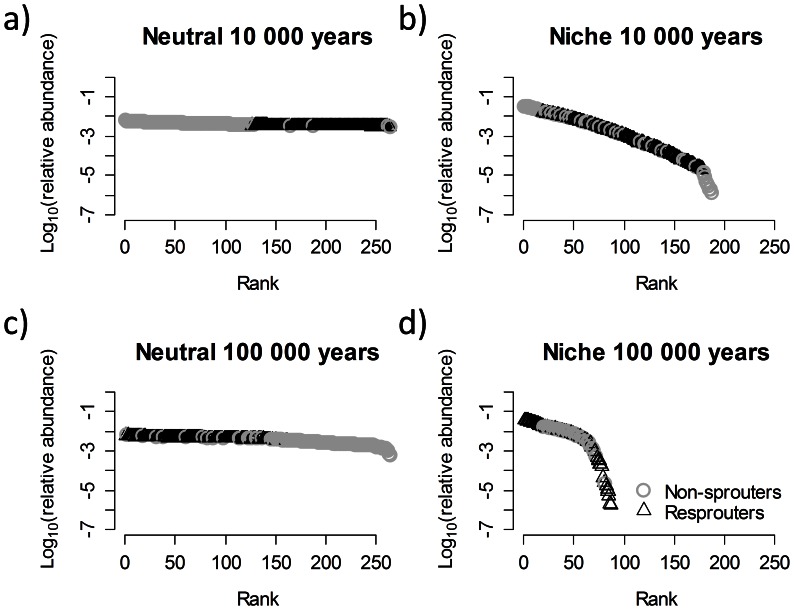
Simulated rank-abundance curves. Simulated rank-abundance curves for two scenarios: neutral scenario, and niche scenario after 10 000 time steps (a, b) and 100 000 time steps (c, d). Circles represent non-sprouting species and triangles resprouting species. The simulation started with 132 non-sprouting and 132 resprouting species.

While observed species richness can be matched for both the neutral model and niche model with density regulation (Fig. 2a,g), their rank-abundance distributions (RAD) differ substantially (Fig. 2c,i). The difference in RAD is more pronounced if the model is initialized with 264 species (Fig. 3). Only the niche model produces structurally realistic RAD as observed in the study area [3], [45], where relative abundances vary over several orders of magnitude and abundances of non-sprouters and resprouters show large within-group variation (Figs. 2 & 3). Using 16 species RAD reached an equilibrium after 10 000 years, whereas the neutral model still largely reflected initial conditions. Results are different for the species-rich system: when 264 species are used, RAD did not reach equilibrium by 100 000 years in the niche-based model, although most of the extinction events occur in the first 50 000 years (not shown here). In the neutral model 100 000 years are not sufficient for realistic differences between species relative abundances to emerge and non-sprouters and resprouters still form relatively homogeneous groups. The temporal dynamics of RADs are presented as animations in the electronic supplementary material (Animations S1 and S2).

### Sensitivity analysis

**Figure 4:**
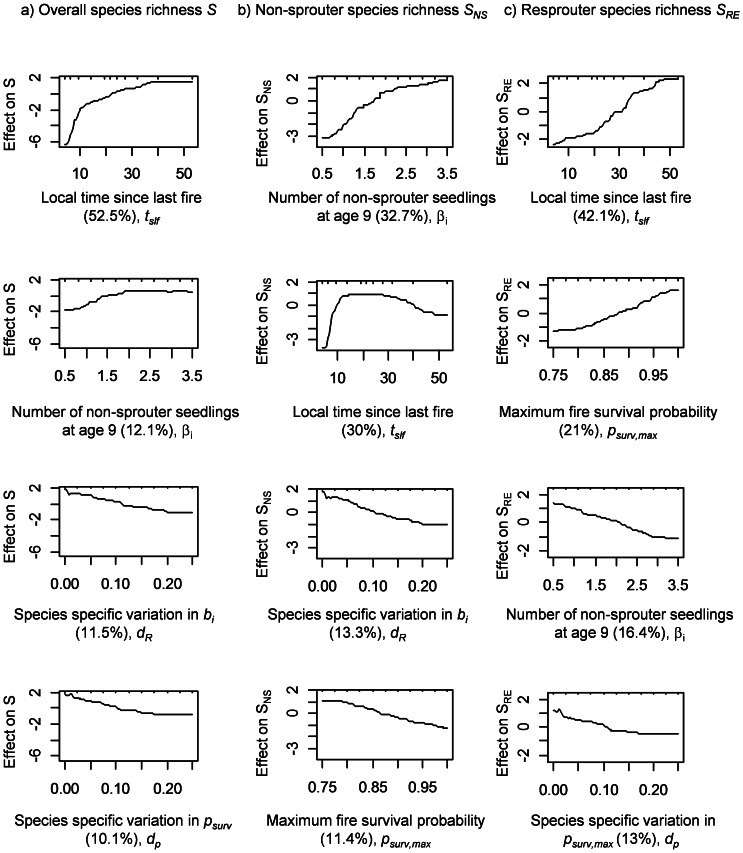
Sensitivity analysis. Partial dependence plots for the four most important predictor variables for: a) overall species richness *S*, b) non-sprouter species richness *S_NS_*, and c) resprouter species richness *S_RE_* based on a boosted regression tree analysis of 2 010 random parameter samples (see Table 1 for details). The fitted value on the y-axis shows the effect of a given variable on the response after accounting for the average effects of all other variables in the model. The relative influence (%) of each predictor variable on the response is given in brackets in the legend of the x-axis.

The relative influence of each parameter, and the average time since last fire, on species richness differed between non-sprouters, *S_NS_*, and resprouters, *S_RE_* (Table 1, Fig. 4). Partial dependence plots are shown for the four most important parameters. These plots are the result of boosted regression tree analysis of simulation runs with 2 010 parameter sets (see Methods section). The most important parameter is the local time since last fire *t_slf_*. Non-sprouter richness *S_NS_* is highest for intermediate fire regimes (15–30 years) while resprouter richness *S_RE_* increases steadily with local time since last fire *t_slf_*. The number of non-sprouter seedlings at age 9 years, β*_i_*, is the second most important parameter influencing species richness and increasing the seedling numbers of non-sprouters has a positive effect on the non-sprouter richness *S_NS_* and a negative impact on resprouter richness *S_RE_* (Fig. 4 and Table 1). The maximum fire survival probability of resprouters *p_surv,max_* is the second most important parameter for resprouter richness *S_RE_*, whereas the impact of this parameter on overall species richness *S* is less important.

## Discussion

The central assumption of neutral theory is that species at the same trophic level are ecologically equivalent [1]. This assumption clearly does not hold for many trophically equivalent species, e.g. fire-killed as opposed to resprouting shrubs in Mediterranean-type fire-prone ecosystems [5], [6]. However, assuming that all species within a single PFT are neutral may help to resolve the apparent paradox that there are far more species than there are ecological niches [36]. We have shown that this approach allows two PFTs, each comprised of completely neutral species, to coexist over a wide range of demographic parameters so long as there is a trade-off between the *per capita* reproductive rate of non-sprouters and the fire survival probability of resprouters. However, and in line with previous studies [7], small departures from the strict neutrality assumption reduce the parameter range across which species may coexist. Therefore, in the absence of stabilizing mechanisms species dissimilarity limits species coexistence. If there is even small interspecific variation, stabilizing processes such as intraspecific density regulation must be operating for species richness to be maintained.

We confirm the importance of the specifics of the fire regime, including time since last fire and mode of fire spread, on species richness [21], [37]. However, in our model, resprouters are more sensitive to changes in the fire regime than are non-sprouters. This differs from previous local community models [38] where non-sprouters were not permitted to compensate for local extinctions by colonization from elsewhere in the metacommunity. Boosted regression tree analysis indicates that non-sprouters are best adapted to intermediate fire regimes (local time since last fire, *tslf* = 12–30 years, which is close to the reported recent fire intervals [39]), whereas resprouters always responded positively to an increase in the time between fires. However, only resprouters can persist under a regime of very frequent fires (e.g. local time since last fire, *tslf* ≤5 years). In the systems we consider, whether fire spread depends more on vegetation age (and therefore on standing biomass) than on weather conditions is contentious [40]–[43]. Our sensitivity analysis showed that fuel age dependency of fire spread has a positive effect on species richness, mainly because it results in a longer average time since last fire [21] (see Fig. S1 in EMS).

Niche and neutral models differ markedly in their temporal dynamics [44]. It has been claimed that if there is a sufficiently large number of species in each niche then niche-based and neutral models produce similar rank-abundance distributions (RAD) [17], [18]. This assertion is not supported by our study. Initial conditions persist in neutral models for a very long time – slow dynamics are fundamental to the maintenance of species richness in such models [1]. If species have different life-history attributes, a distinctive community structure emerges much faster. After 100 000 years, only those models that included species-specific variation resulted in RAD close to those observed near our study site [3], [45]. Although it has been shown that neutral models can be parameterized for Mediterranean-type ecosystems [2], [3], the slow transient dynamics revealed in our simulation model and the empirical study of Perry et al. [3] cast some doubt on their suitability.

Theoretical studies have shown that identical species cannot stably coexist in a network of habitat patches [46]. However, it is questionable whether an equilibrium perspective is appropriate for ecosystems where environmental conditions can change substantially over the lifespan of long-lived individuals [47], [48]. This is why we focus on transient system dynamics over a period of 10 000 years (equivalent to 200–2000 generations of non-sprouters). We know much less about the dynamics of the neutral model than we do about its steady-state predictions [49]. Shifting focus from equilibrium steady-state to transient dynamics [50] suggests that similar species can coexist over ecological time-scales in niche-based models similar to that of Wang, Zhang and Wang [46]. Exploring transient dynamics with simulation models is important if the response of ecological systems is delayed or memory effects are present [51], [52]. On the other hand, simulation models often comprise a large set of parameters and a number of processes and rules, and it is a non-trivial task to investigate the impact of individual parameters on the results and to disentangle the influence and interactions of different processes [53]. Therefore thorough and rigorous sensitivity analyses are crucial for such models [53].

Despite the importance of sensitivity analyses, coping with the structural complexity and number of parameters in detailed models is technically demanding. Modern statistical computational methods such as boosted regression trees [33], together with new modelling strategies such as pattern-oriented modelling [54], provide us with powerful tools to deal with high-dimensional problems. The combination of stochastic process-based simulation models and flexible model analysis tools will enable us to improve our understanding of the processes that maintain species diversity beyond the lessons we have learned from deterministic equation-based models.

## Conclusions

Combining the niche and neutral approaches by creating niche-based models for PFTs yielded a metacommunity model that allowed the long-term coexistence of non-sprouting and resprouting species in fire-prone Mediterranean-type shrublands across a wide range of values for several key demographic parameters. Coexistence of these two PFTs could be explained by a trade-off between the higher reproductive rate of non-sprouters and fire survival probability of resprouters. Species richness was maintained under two contrasting assumptions: i) species within one PFT are identical and intraspecific density regulation is not required, or ii) species show specific variation in their demographic attributes and intraspecific density regulation is required. However, rank-abundance distributions in the neutral model remain dominated by initial conditions even after 100 000 years because only chance events structure the community. In contrast, the niche-based approach, where species within a particular PFT show interspecific variation, together with intraspecific density regulation, resulted in a more realistic rank-abundance structure over the same time period. Our results emphasise the ecological importance of variability in species characteristics, and demonstrate that neglecting this variation can have a large impact on our understanding of system dynamics that might lead to an underestimate of extinction risks for rare species.

## Supporting Information

Table S1 **Scenario overview.** Overview of the three scenarios presented and discussed in the main text. Scenarios are characterised by the range of species-specific variation in number of seedlings per adult (*d_R_* ) and the strength of density regulation. (*f_K_^-1^*). In all three scenarios fire survival does not differ between species (*d_p_* = 0) and fire spread does not depend on vegetation age (*f_mode_* = 0).
(DOCX)

Figure S1 **Time since last fire.** Average time since last fire of habitat grid cells is a function of the basic fire-spread probability *p_c_* and the mode of fire spread *f_mode_*, i.e. whether fire spread depends on the fuel age (filled circles) or not (unfilled circles).
(TIF)

Methods S1 **Details – description of the submodels.**
(DOCX)

Animation S1 **Temporal dynamics of RADs for the neutral scenario.** Corresponding to Figure 3 a, we present the temporal dynamics of the RAD for the neutral scenario initialized with 132 non-sprouter species (circles) and 132 resprouter species (triangles) over 10 000 years.
(MP4)

Animation S2 **Temporal dynamics of RADs for the niche scenario.** Corresponding to Figure 3 b, we present the temporal dynamics of the RAD for the niche scenario initialized with 132 non-sprouter species (circles) and 132 resprouter species (triangles) over 10 000 years.
(MP4)
