# Supplementary material for: Species-Specific Traits plus Stabilizing Processes Best Explain Coexistence in Biodiverse Fire-Prone Plant Communities
Source: PLoS One. 2013 May 29;8(5):e65084. doi: 10.1371/journal.pone.0065084 (PMC3667055; doi:10.1371/journal.pone.0065084)
Supplement: Table S1 — Scenario overview. Overview of the three scenarios presented and discussed in the main text. Scenarios are characterised by the range of species-specific variation in number of seedlings per adult (dR ) and the strength of density regulation. (fK-1). In all three scenarios fire survival does not differ between species (dp = 0) and fire spread does not depend on vegetation age (fmode = 0). (DOCX) [file pone.0065084.s001.docx]

| Scenario description | *d_R_* | *f_K_^-1^* |
| --- | --- | --- |
| Neutral | 0 | 0 |
| Non-neutral | 0.1 | 0 |
| Niche | 0.1 | 1 |
