# Supplementary figures and images for: Species-Specific Traits plus Stabilizing Processes Best Explain Coexistence in Biodiverse Fire-Prone Plant Communities
Source: PLoS One. 2013 May 29;8(5):e65084. doi: 10.1371/journal.pone.0065084 (PMC3667055; doi:10.1371/journal.pone.0065084)

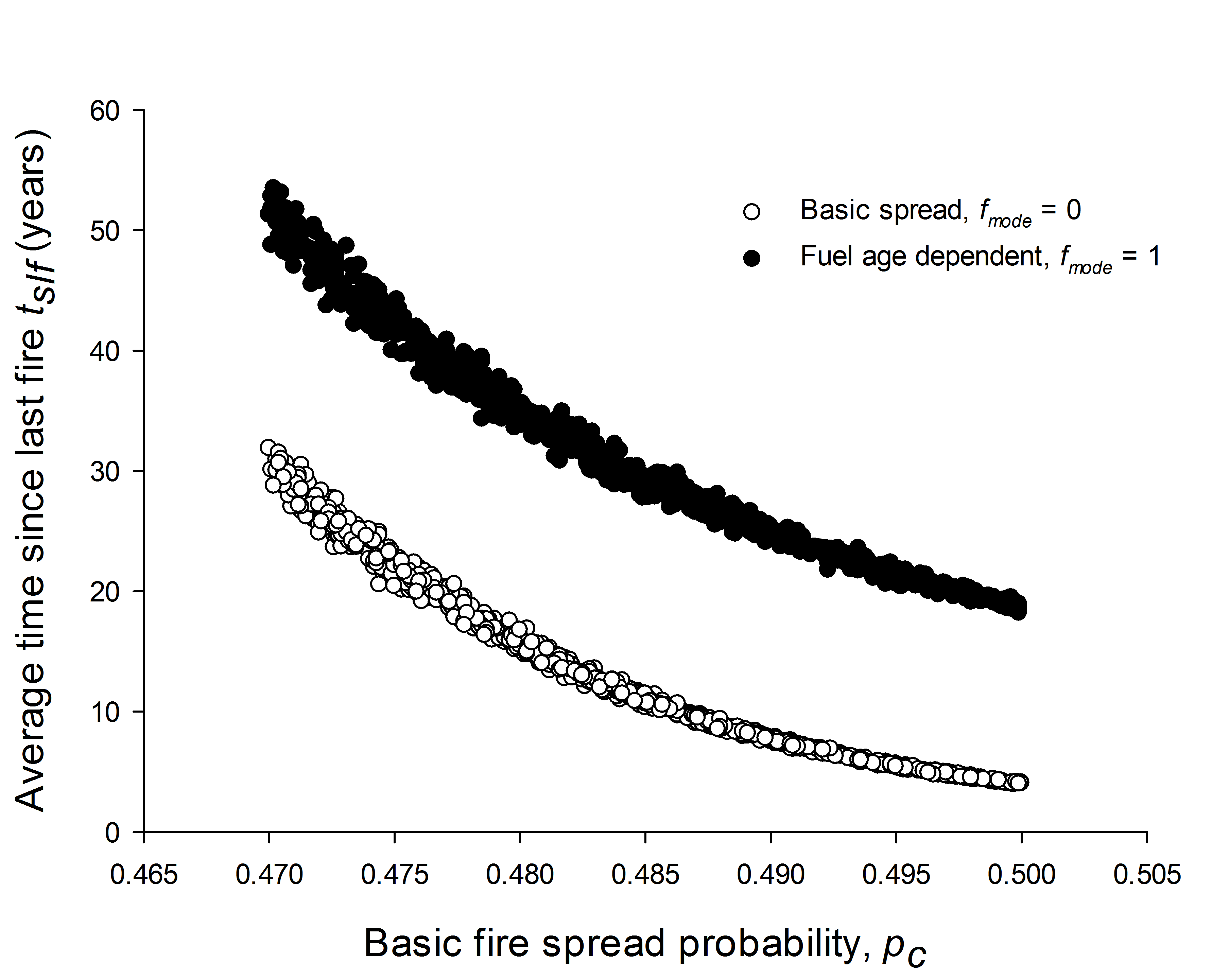

Supplement: Figure S1 — Time since last fire. Average time since last fire of habitat grid cells is a function of the basic fire-spread probability pc and the mode of fire spread fmode, i.e. whether fire spread depends on the fuel age (filled circles) or not (unfilled circles). (TIF) [file pone.0065084.s002.tif]
