# Supplementary material for: Species-Specific Traits plus Stabilizing Processes Best Explain Coexistence in Biodiverse Fire-Prone Plant Communities
Source: PLoS One. 2013 May 29;8(5):e65084. doi: 10.1371/journal.pone.0065084 (PMC3667055; doi:10.1371/journal.pone.0065084)
Supplement: Methods S1 — Details – description of the submodels. (DOCX) [file pone.0065084.s003.docx]

Methods S1: *Details – description of the submodels:*

Fire spread

During each time step (one year) one randomly selected grid cell is ignited. Fire spreads with probability *p_spread_* to that cell’s four nearest neighbours. Fire spread continues from all ignited grid cells in the same way. Grid cells that have been ignited can only burn once in a given fire. This process continues until all grid cells have been burnt or fire spread ceases (i.e. no new cells are ignited). Fire-spread probability, *p_spread_*, can either be constant from year-to-year, *p_c_*, or can depend on the local time since last fire, *t*, as a proxy for the available fuel load [23]: *p_spread_* = *p_c_* × {1 + exp[-*d* × (*t - b*)]}, where *d* determines the steepness of the sigmoidal curve and *b* parameterizes the point in time when half of the basic fire-spread probability *p_c_* is reached. The parameterization is chosen to reflect the fact that fire-spread probability is low when the time since last fire is less than four years (e.g. *p_spread_* = 0.12 × *p_c_,* for *t* = 3 years), increases rapidly to about eight years (*p_spread_* = 0.95× *p_c_,* for *t* = 8 years) and more slowly thereafter. For the same constant spread probability, *p_c_*, the dependency on fuel load results in an increase of the average time since last fire *t_slf_* for about 10–20 years (see Fig. S1).

*Seed dispersal and seed production*

When a grid cell burns, the canopy seed stores for all plants within it are released and all non-sprouters are killed and a fraction of resprouters survive (see fire survival for details). We model seedlings rather than seeds to avoid very large seed numbers. The number of seedlings per adult *b_i_* (*i* indicates the species) that are dispersed after a fire for non-sprouters depends on the time since last fire and for resprouters on the age of the plant and the local time since last fire, based on detailed studies of the demography of large serotinous shrubs at Eneabba [25—27]. In table 1 and 2 at the end of this document we present the number of seedlings per adult *b_i_* for non-sprouters and resprouters. New non-sprouters cannot store viable seeds until they reach four years of age. Number of seedlings produced per adult for non-sprouters (*b_i_*) reaches its maximum at 20–25 years [25] and then slowly declines, reflecting increased annual interfire mortality as shrubs approach their maximum longevity (Fig. 1b). Thus, *b_i_* combines two processes: decreasing overall stand density due to interfire mortality, and seed accumulation over time. The number of seedlings (*S*) generated by a local population (for resprouters cohort) after a fire is then determined by the product of the local population (for resprouters cohort) size after the last fire (*N*) and the number of seedlings each adult produces, *b_i_* (*S* = *N* × *b_i_)*. For non-sprouters, where all adult shrubs are killed, *b_i_* is the potential local population growth rate. However, the realised growth rate depends on the number of sites available for establishment and interspecific competition, and for resprouters on the fire survival of shrubs. Resprouters require longer to attain a positive *b_i_* (30 years; [19]). After resprouters have reached reproductive maturity they produce seeds but, on average, fewer than non-sprouters [22]. Although the maximum number of seedlings per adult *b_i_* is approximately 0.6 for resprouters their population can grow given a sufficient fire survival of the mature shrubs. A mature resprouter affected by fire recovers and recommences seed production three years after fire. The number of seedlings produced per resprouter increases with time since last fire (reflecting an accumulating canopy seed bank) until time since last fire exceeds 15 years, after which it is constant (Fig. 1b). For resprouters we ignore interfire mortality, which is very low [27], and only model fire mortality (Fig. 1c).

Number of seedlings per adult *b_i_* can depend on species (correction factor for species *i*, *a_i_)*, and on intraspecific density regulation (expressed by the parameter *f_K_* that describes the strength of the density regulation). The number of seedlings *b_i_* is multiplied by a factor β_i_ (seedling modification factor, reference value that is used in the simulations if not mentioned otherwise β_i_ = 1.8). The parameter β_i_ can also be interpreted as the number of non-sprouter seedlings at age 9 since *b_i_* = 1 for time since last fire *tslf* = 9 years. For resprouters β_i_ is always 1. Whenever a patch burns all individuals disperse their seeds, and the adjusted number of seedlings per adult *b_i,adj_* is given by:

$b_{i,adj}\left(tslf,age\right)=a_{i}\cdot\beta_{i}\cdot b_{i}\left(tslf,age\right)\cdot\left(1\text{-}\frac{N_{i}}{f_{K}\cdot n\cdot K}\right)$ (1)

Values for *b_i_* are looked up in tables based on detailed studies of the demography of large serotinous shrubs at Eneabba [21, 25—27]. The modifying factors of the number of seedlings per adult *b_i_* are calculated as follows: Species-specific differences in growth rates are parameterized by a maximum relative difference between the lowest and the highest seedling numbers. All other species-specific number of seedlings *b_i_* are evenly distributed between the extreme values. Therefore, *n* evenly spaced species-specific factors *a_i_* (0 ≤ *a_i_ < ∞)* subdivide the interval [1 - *d_R_*/2,1 + *d_R_*/2] symmetrically around 1 (if possible), otherwise the interval [0,*d_R_*] is used to avoid negative *a_i_*’s, in (*n* – 1) intervals of equal length. Finally, the adjusted number of seedlings *b_i,adj_* can also be influenced by intraspecific competition, expressed by a density-dependent term (1 *- N_i_*/(*f_K_* × *n* × *K*)), where *N_i_* is the abundance of species *i* in the patch*, f_K_* (0 < *f_K_* < ∞) modifies the intraspecific density regulation (and 1/*f_K_* is the strength of the intraspecific density regulation)*,* *n* is the number of grid cells in the patch, and *K* is the grid cell’s carrying capacity (approximately 65 000 individuals). If the term (1 *- N_i_*/(*f_K_* × *n* × *K*)) results in negative values we set its value to zero. Interspecific competition is considered in the establishment process, but is not considered in the determination of the number of seedlings (see Establishment).

When seedlings are released, a fraction *m* establishes in the wider metacommunity with the majority of seedlings (1 - *m*) remaining in the patch. All seedlings that remain in the patch are evenly distributed across the burnt grid cells of the home patch. All seedlings dispersed into the metacommunity are evenly distributed across all burnt grid cells in the landscape. This is a reasonable simplification at the spatial scale we focus on (4 *×* 4 km) given the frequent long-distance dispersal events of several kilometres reported for this system [20]. In the following we often refer to the number of non-sprouter seedlings at age 9 years, which is identical to the seedling modification factor β_i_. If β_i_ = 1 it takes non-sprouters 9 years to have a seed store sufficient to replace themselves after fire.

*Fire survival of resprouters*

Resprouters survive fire with an age-dependent basic survival probability *p_survb_* (Fig. 1c), with survival increasing linearly from 0.1 at an age of one year to a maximum survival probability of 0.99 at an age of 10 years. Survival probability decreases linearly with age from 199 years to zero at age 299 (maximum longevity, [27]). Fire survival can be adjusted by the fire survival modification factor h (0 < h < 1). In the paper we refer to the maximum fire survival probability, that is *p_surv,max_* = h × p_survb_ (for mature resprouters and time since last fire 10 ≤ *tslf* ≤ 199). Fire survival probability may vary in the same way as the number of seedlings per adult *b_i_* between species *g_i_.* Thus, the basic survival probability *p_survb_* is modified as follows:

$p_{surv,i}=g_{i}\cdot h\cdot p_{survb}$ (2)

Interspecific variation in survival, *g_i_*, is modelled such that species specific maximum survival probabilities *p_surv,max,i_* are a sequence of evenly spaced values between (*p_surv,max_* - *d_p_*/2,*p_surv,max_* + *d_p_*/2) when (*p_surv,max_* + *d_p_*) *≤* 1 and the interval (1 - *d_p_*,1) otherwise. The modification factor *g_i_* is then *g_i_* = *p_surv,max,i_*/*p_surv,max_*. The maximum difference between maximum fire survival probabilities is *d_p_*. Fire survival of resprouters is binomially distributed with a probability of *p_surv,i_*. If the product of the number of individuals *n* and the fire survival probability, *p_surv,i_*, is ≥ 5.5 and [*n ×* (1 - p*_surv,i_*)] ≤ 5.5, the demographic variability can be neglected and the number of survivors approximated by the mean of the respective binomial distribution (µ = *n* × *p_surv,i_*).

*Establishment*

When a grid cell is burnt all non-sprouters and a fraction of resprouters, depending on the fire survival probability, die. These empty spaces can be colonized by the seedlings of burnt (killed and resprouting) plants originating either from the patch itself or from other burnt patches within the metacommunity (see *Seed dispersal and seed production section)*. If the number of seedlings is less than or equal to the number of available sites in a grid cell then all the seedlings establish. To represent demographic variation at the grid cell level the number of new recruits is determined by drawing, for each species *i*, a random number from a Poisson distribution, where the mean equals the number of seedlings for species *i*. If the number of seedlings exceeds the number of available sites in a grid cell (*K_gridcell_* minus surviving resprouters), the successful individuals are determined by lottery competition, i.e. are drawn from a multinomial distribution using the relative abundances as probabilities.

Table 1: basic number of seedlings per adult *b_i_* for non-sprouters. The age of non-sprouters is always the same as the time since last fire. The table is also visualized in Figure 1b).

| Time since last fire | Basic number of seedlings per adult *b_i_* |
| --- | --- |
| 1 | 0.00E+00 |
| 2 | 0.00E+00 |
| 3 | 0.00E+00 |
| 4 | 2.32E-01 |
| 5 | 3.04E-01 |
| 6 | 4.06E-01 |
| 7 | 5.45E-01 |
| 8 | 7.35E-01 |
| 9 | 1.00E+00 |
| 10 | 1.35E+00 |
| 11 | 1.78E+00 |
| 12 | 2.32E+00 |
| 13 | 2.94E+00 |
| 14 | 3.62E+00 |
| 15 | 4.32E+00 |
| 16 | 5.00E+00 |
| 17 | 5.60E+00 |
| 18 | 6.09E+00 |
| 19 | 6.46E+00 |
| 20 | 6.71E+00 |
| 21 | 6.86E+00 |
| 22 | 6.93E+00 |
| 23 | 6.93E+00 |
| 24 | 6.88E+00 |
| 25 | 6.81E+00 |
| 26 | 6.64E+00 |
| 27 | 6.39E+00 |
| 28 | 6.07E+00 |
| 29 | 5.71E+00 |
| 30 | 5.30E+00 |
| 31 | 4.87E+00 |
| 32 | 4.42E+00 |
| 33 | 3.97E+00 |
| 34 | 3.52E+00 |
| 35 | 3.09E+00 |
| 36 | 2.68E+00 |
| 37 | 2.30E+00 |
| 38 | 1.94E+00 |
| 39 | 1.63E+00 |
| 40 | 1.35E+00 |
| 41 | 1.10E+00 |
| 42 | 8.88E-01 |
| 43 | 7.07E-01 |
| 44 | 5.57E-01 |
| 45 | 4.33E-01 |
| 46 | 3.32E-01 |
| 47 | 2.51E-01 |
| 48 | 1.88E-01 |
| 49 | 1.38E-01 |
| 50 | 1.01E-01 |
| 51 | 7.21E-02 |
| 52 | 5.10E-02 |
| 53 | 3.55E-02 |
| 54 | 2.44E-02 |
| 55 | 1.65E-02 |
| 56 | 1.10E-02 |
| 57 | 7.24E-03 |
| 58 | 4.68E-03 |
| 59 | 2.98E-03 |
| 60 | 1.87E-03 |
| 61 | 1.15E-03 |
| 62 | 7.01E-04 |
| 63 | 4.18E-04 |
| 64 | 2.46E-04 |
| 65 | 1.42E-04 |
| 66 | 8.03E-05 |
| 67 | 4.47E-05 |
| 68 | 2.45E-05 |
| 69 | 1.31E-05 |
| 70 | 6.93E-06 |
| 71 | 3.58E-06 |
| 72 | 1.82E-06 |
| 73 | 9.02E-07 |
| 74 | 4.39E-07 |
| 75 | 2.10E-07 |
| 76 | 9.79E-08 |
| 77 | 4.47E-08 |
| 78 | 2.00E-08 |
| 79 | 8.74E-09 |
| 80 | 3.73E-09 |
| 81 | 1.56E-09 |
| 82 | 6.33E-10 |
| 83 | 2.51E-10 |
| 84 | 9.73E-11 |
| 85 | 3.67E-11 |
| 86 | 1.35E-11 |
| 87 | 4.81E-12 |
| 88 | 1.67E-12 |
| 89 | 5.62E-13 |
| 90 | 1.84E-13 |
| 91 | 5.83E-14 |
| 92 | 1.79E-14 |
| 93 | 5.31E-15 |
| 94 | 1.52E-15 |
| 95 | 4.22E-16 |
| 96 | 1.13E-16 |
| 97 | 2.90E-17 |
| 98 | 7.16E-18 |
| 99 | 1.70E-18 |
| 100 | 3.85E-19 |
| >100 | 0.00E+00 |

Table 2: basic number of seedlings per adult *b_i_* for resprouters. If resprouters are younger than 30 years they do not produce any seeds. If they are older than 30 years their basic number of seedlings per adult *b_i_* depends on the time since last fire. If the onset of maturity started within the last inter fire interval the time since last fire has to be corrected accordingly, i.e. for example if a plant is 35 years of age and the last fire has happened 10 years before the value for time since last fire = 5 is chosen. The table is also visualized in Fig. 1b.

| Time since last fire | Basic number of seedlings per adult *b_i_* | |
| --- | --- | --- |
| 1 | 0.00 |  |
| 2 | 0.00 |  |
| 3 | 0.02 |  |
| 4 | 0.07 |  |
| 5 | 0.13 |  |
| 6 | 0.20 |  |
| 7 | 0.28 |  |
| 8 | 0.35 |  |
| 9 | 0.41 |  |
| 10 | 0.46 |  |
| 11 | 0.50 |  |
| 12 | 0.54 |  |
| 13 | 0.57 |  |
| 14 | 0.59 |  |
| 15 | 0.61 |  |
| >15 | 0.61 |  |
